# Fixed drug eruption resulting from fluconazole use: a case report

**DOI:** 10.4076/1752-1947-3-7368

**Published:** 2009-07-06

**Authors:** Mahkam Tavallaee, Mahnaz Mahmoudi Rad

**Affiliations:** 1 Department of Health Sciences, Simon Fraser University, Burnaby, Canada V5A 1S6; 2 Skin Research Center, Shaheed Beheshti University of Medical Sciences and Health Services, Tehran, Iran

## Abstract

**Introduction:**

Fluconazole is a widely used antifungal agent with a possible side effect of fixed drug eruption. However, this adverse drug effect is absent from the reported list of possible side effects of fluconazole. We are presenting a rare case in our report.

**Case presentation:**

A 25-year-old Iranian woman developed fixed drug eruptions on different sites of her body after taking five doses of fluconazole to treat vaginal candidiasis. A positive patch test, positive oral challenge test and skin biopsy were all found to be consistent with fixed drug eruption.

**Conclusion:**

Fluconazole is a widely prescribed drug, used mainly to treat candidiasis. Fixed drug eruption as a possible side effect of Fluconazole is not well known and thus, the lesions may be misdiagnosed and mistreated. Based on our findings, which are consistent with a number of other practitioners, we recommend adding fixed drug eruption to the list of possible side effects of fluconazole.

## Introduction

Fluconazole (Diflucan®) is a broadly used bis-triazole antifungal agent. It functions mainly by inhibiting cytochrome P45014a-demethylase (P45014DM), which in turn, prevents the conversion of lanosterol to ergosterol in the sterol biosynthesis pathway. Fluconazole seems to have a superior selectivity for fungus compared to human P-450-enzymes [1]. It is used to treat vaginal, oropharyngeal, and esophageal candidiasis; additionally, it is used to treat cryptococcal meningitis and prevent fungal infections in immunocompromised patients. In some literature, fluconazole has proven to be effective for treatment of Candida urinary tract infections, peritonitis, and systemic Candida infections including candidemia, disseminated candidiasis, and pneumonia [2]. The body clears fluconazole primarily by renal excretion. Accordingly, the dosage should be reduced in proportion to any reduction in kidney function. In general, the body tolerates fluconazole quite well. Still, there are commonly observed adverse events such as nausea, vomiting and elevations in liver function tests. Less common side effects are headaches, dizziness, diarrhea, stomach pain, heartburn, change in ability to taste food, an upset stomach, extreme fatigue, appetite loss, pain in the right upper quadrant, jaundice, dark urine, pale stools, flu-like symptoms and seizures. Observed hypersensitivity reactions are anaphylactic reactions, angioedema and facial edema, pruritus, urticaria, erythematous or maculopapular rash and exfoliative skin reactions, including Stevens Johnson Syndrome (SJS) and toxic epidermal necrolysis [3].

Fixed drug eruption (FDE) is characterized by single or multiple skin lesions that occur at the same site each time a drug is administered. However, the number and size of sites may increase after each exposure. Lesions are usually round or oval and well defined. Swelling and redness of skin are typically seen within 30 minutes to eight hours after exposure. Lesions are more commonly seen in the extremities, genital areas and perianal areas, and may also appear in other locations such as the mucosal area. Persistent hyperpigmentation on the site of the lesion is normally seen after healing. Accompanying systemic symptoms are mild in FDE. The drugs mostly reported to cause FDE are: cotrimoxazole, tetracycline, metamizole, phenylbutazone, paracetamol, acetylsalicylic acid, NSAIDS, metronidazole, tinidazole, chlormezanone, amoxicillin, ampicillin, erythromycin, belladonna, griseofulvin, phenobarbitone, diflunisal, pyrantel pamoate, clindamycin, allopurinol, orphenadrine, albendazole, dapsone, phenolphthalein, oral contraceptives, phenacetin, doxycycline, minocycline, panmycin, sulfonamide, sulfasalazine, benzodiazepines and chlordiazepoxide, hyoscine butylbromide, and quinine [3,4].

## Case presentation

A 25-year-old woman received five doses of fluconazole (150 mg) once a month for recurrent vaginal candidiasis. She was healthy but had a family history of atopic dermatitis. She noticed a red erythematous macule on the medial side of her right popliteal fossa after taking her second dose of fluconazole. With time, the macule faded, but a violet pigmentation developed. A month later, after taking another dose, she again developed two macules; one developed on exactly the same site and the other in the left popliteal fossa. Both patches were symmetrical and similar in appearance. Again, the patches faded and hyperpigmented areas developed. At this point, she was examined by a dermatologist and misdiagnosed with lichen planus and treated with topical clobetasol that led to the development of striae on both sites. Four hours after taking the fifth dose, the macules reappeared along with a new macule on her right upper lip. She suspected that the symptoms were caused by fluconazole and again visited a dermatologist. An oral challenge test with fluconazole (150 mg) was conducted 4 weeks later and showed similar signs three hours after intake. Local provocation was performed with 10% fluconazole in petrolatum on the left pigmented area and 10% fluconazole in ethanol on the right pigmented area. For comparison, the same compounds were tested on normal skin on the back. After 16 hours, two red patches developed on both sides of her legs and none on her back. A skin biopsy specimen from the left popliteal area revealed a lichenoid infiltrate, a basal cell vacuolization, dermal melanophages and a superficial perivascular lymphocytic infiltrate consistent with FDE. Despite having a family history of atopic dermatitis, she had no major or minor symptoms of atopic dermatitis and she denied having any other reaction to drugs or any allergy history. We recommended that she discontinue using fluconazole.

To determine the cause of the recurrent vaginitis, her complete medical history was taken. She had experienced mid cycle spotting while using low-dose oral contraceptives therefore she switched to using high-dose oral contraceptives 2 years before our study. She was also working in a fitness center during that time and she mentioned that she wore a wet swimsuit for long periods of time and used tight synthetic clothes all of which were risk factors for vaginal candidiasis [16]. At this time, we recommended that she use other contraceptive methods for example, condoms. Furthermore, we asked her to wear cotton underwear and informed her of preventive methods of candidiasis. We followed up with her after 6 months and she mentioned she had not experienced any other episode of candidiasis since then.

## Discussion

We discuss a rare case of fixed drug eruption due to fluconazole. We have found 16 other reports of FDE due to fluconazole although the site and appearance of the skin lesions were different among the reported cases. Thirteen out of 17 cases of FDE due to fluconazole, including ours, occurred in women [6,8]-[10,12]-[14]. Most previous studies on FDE due to drugs demonstrated a higher occurrence in men compared to women according to research by Mahboob and colleagues [4]. Contrarily, in their study, the ratio of women to men was 1.1:1 [4]. The number of reported cases of FDE due to fluconazole is too low to be discussed epidemiologically. However, the higher ratio of women could be due to the higher prescription of fluconazole to women due to vaginal candidiasis. The youngest patient reported was 19-years-old [11] while the oldest one was 66-years-old [7]. The mean age ± SD of men who experienced FDE due to drugs in previous studies was 30.4 ± 17 while it was 31.3 ± 14 for women [4]. In previous studies of FDE due to fluconazole, all female patients were prescribed fluconazole for vaginal candidiasis similar to our patient, while male patients were prescribed fluconazole for Candida balanitis [5], oral candidiasis [10], tinea corporis and tinea cruris [11]. In almost all cases, eruption occurred after a couple of drug administrations. The only common medication used by patients was fluconazole. The most affected sites for eruptions were limbs, palmar and plantar areas [5,6,8,10,12] as well as the oral cavity and lips [6,8,9,11,14,15]. A report of vulvar FDE due to fluconazole has also been published recently [15]. In our patient, both popliteal fossas and the upper lip were affected.

A study by Sharma and colleagues was conducted on 125 patients who had FDE due to drugs. They reported that the trunk and limbs were the major sites with 24% followed by lips alone and genitalia with 20.8% and 20%, respectively. Another study compared 450 cases of FDE and the results showed that 48% of lesions involved the lips followed by hands (36.8%), arms (34.6%), and legs (33.3) [4]. They also reported that 57.6% of patients had multiple lesions compared to 42.4% who had a single eruption due to use of drugs [17]. In a study conducted by Mahboob et al, 16.2% of patients with FDE had solitary lesions compared to the remaining patients who had more than one lesion [4]. The same study also showed that 70% of patients with FDE experienced a bilateral involvement comparable to our study [4]. To our knowledge, our study is the second documented case of successful patch testing for fluconazole [6]. Alanko conducted a topical provocation test on 30 patients with FDE and concluded that the test is a useful and safe method. However, a positive test is more informative than a negative test in most drug reactions [18]. One study demonstrated that an oral challenge test is a useful method as a tolerance test or to exclude drug hypersensitivity in cases of suspected drug reactions with negative skin tests [19]. The skin test was positive in our patient. However, we did an oral test as well that was also positive. This was consistent with most of the reported cases [5,7,8,10,11,14]. It is important to note that diagnostic tests must be done carefully to prevent the rare occurrence of toxic epidermal necrolysis [13]. Skin biopsies were performed in four cases [5,6,12,14] and the histopathology was consistent with FDE similar to our patient.

## Conclusion

Fluconazole is a broadly administered medication used mainly to treat candidiasis. FDE is one of the rare side effects of fluconazole. However, FDE may be misdiagnosed and mistreated since many medical practitioners are unaware of this uncommon side effect. Our findings are consistent with those from a number of other practitioners, thus, we recommend adding FDE to the list of possible side effects of fluconazole.

**Figure 1:**
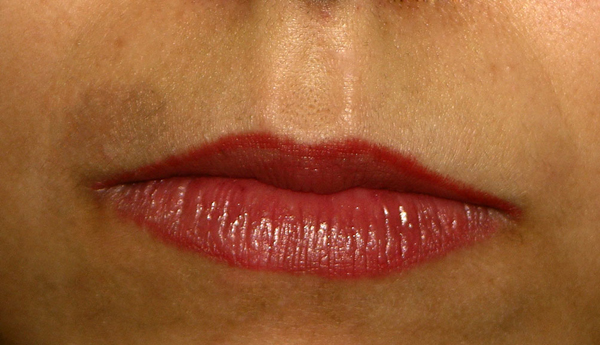
**Fixed drug eruption induced by fluconazole, upper lip area**.

**Figure 2:**
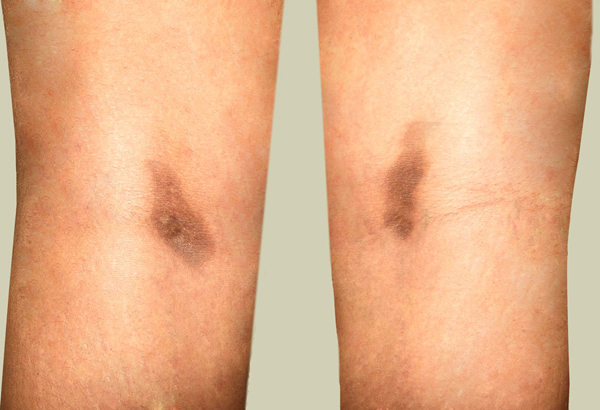
**Symmetrical fixed drug eruption on legs due to fluconazole**.

**Figure 3:**
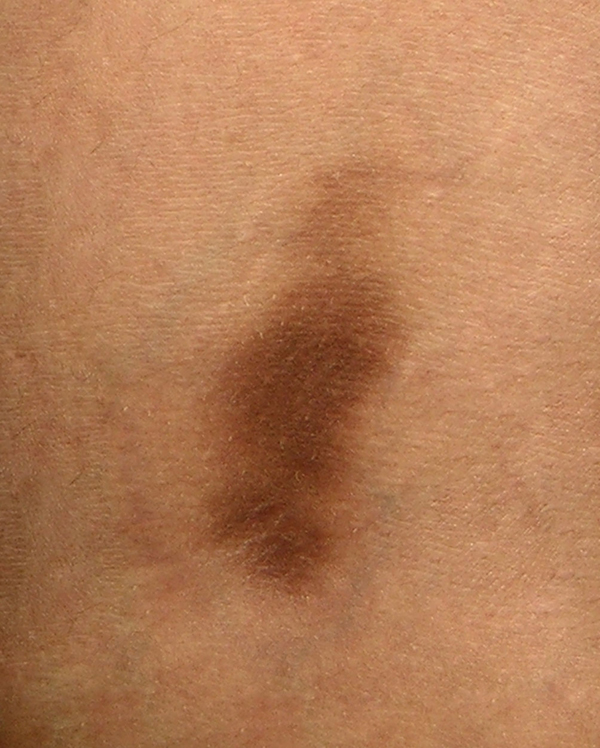
**Fixed drug eruption due to fluconazole (right popliteal area)**.

**Figure 4:**
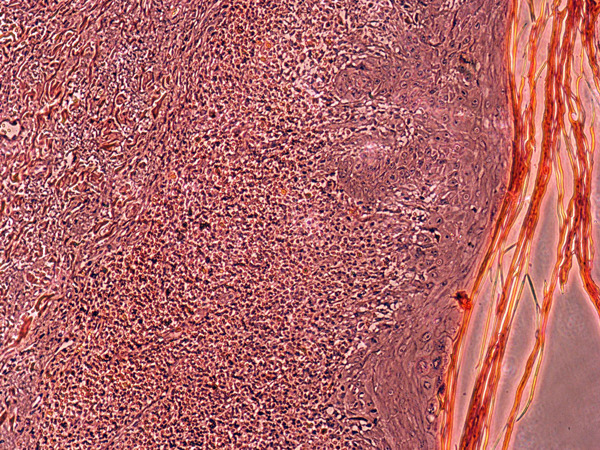
**Histopathology of skin biopsy (left popliteal area)**.

**Figure 5:**
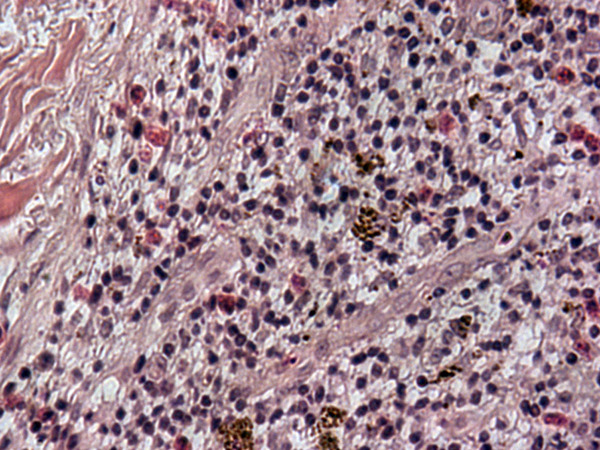
**Histopathology of skin biopsy (left popliteal area)**.

## Abbreviations

FDE: fixed drug eruption; P45014DM: P45014a-demethylase; SJS: Stevens Johnson Syndrome.

## Consent

Written informed consent was obtained from the patient for publication of this case report and any accompanying images. A copy of the written consent is available for review by the Editor-in-Chief of this journal.

## Competing interests

The authors declare that they have no competing interests.

## Authors' contributions

MT identified the adverse drug reaction, performed the literature review and wrote the first draft of the paper. NMR took the biopsy, undertook the histological analysis and finalized the manuscript. Both authors read and approved the final manuscript.
